# Botanical Society of Edinburgh

**Published:** 1836-10

**Authors:** 


### BOTANICAL SOCIETY OF EDINBURGH.

THIS Society, which is exclusively devoted to the advancement of Botanical Science, was instituted the 17th of March last. Its peculiar feature, and that in which non-residents are peculiarly interested, is the provision made for the interchange of dried specimens. "Desiderata and duplicates will, as far as possible, be supplied from the Society's collections to all its members; and individuals, anxious to improve or complete herbaria, will thus secure the important advantage of exchanging the botanical productions of their respective districts for those of others more remotely situated. The Flora of Edinburgh, which is particularly rich, will afford a constant supply of valuable duplicates, and many rare species will be annually obtained from the mountainous parts of Scotland." The following are some of the laws of the Society which apply to non-resident Members: 1. Any person not residing in Edinburgh may be elected a non-resident member, on being recommended by two members of any scientific or literary society, and paying 2*l.* 2*s.* 2. Any person residing abroad may be admitted a foreign member, on the transmission of five hundred specimens, (including at least one hundred species,) or a botanical work of which he is the author: if the former, he will be entitled to specimens from the Society's collection n exchange. 3. To entitle a resident or non-resident member to a share of the Society's duplicates, it is requisite that he shall not be in arrear, and shall have given to the Society, in the course of the year, not less than fifty specimens of plants.

The members are to be called "Fellows of the Botanical Society."

Edinburgh is in every respect well suited for the head-quarters of such a society: its Flora is both rich and rare; its botanical garden is admirably arranged and regulated; and its professor (whom we rejoice to see as president of this institution,) is eminently qualified to communicate to a numerous class of pupils, collected for a time from all parts of the globe, that enthusiasm for botany which he himself possesses, and without which no progress can be made in the science. We heartily wish it success.

